# Supplementary figures and images for: Effects of portable air cleaners and A/C unit fans on classroom concentrations of particulate matter in a non-urban elementary school
Source: PLoS One. 2022 Dec 1;17(12):e0278046. doi: 10.1371/journal.pone.0278046 (PMC9714748; doi:10.1371/journal.pone.0278046)

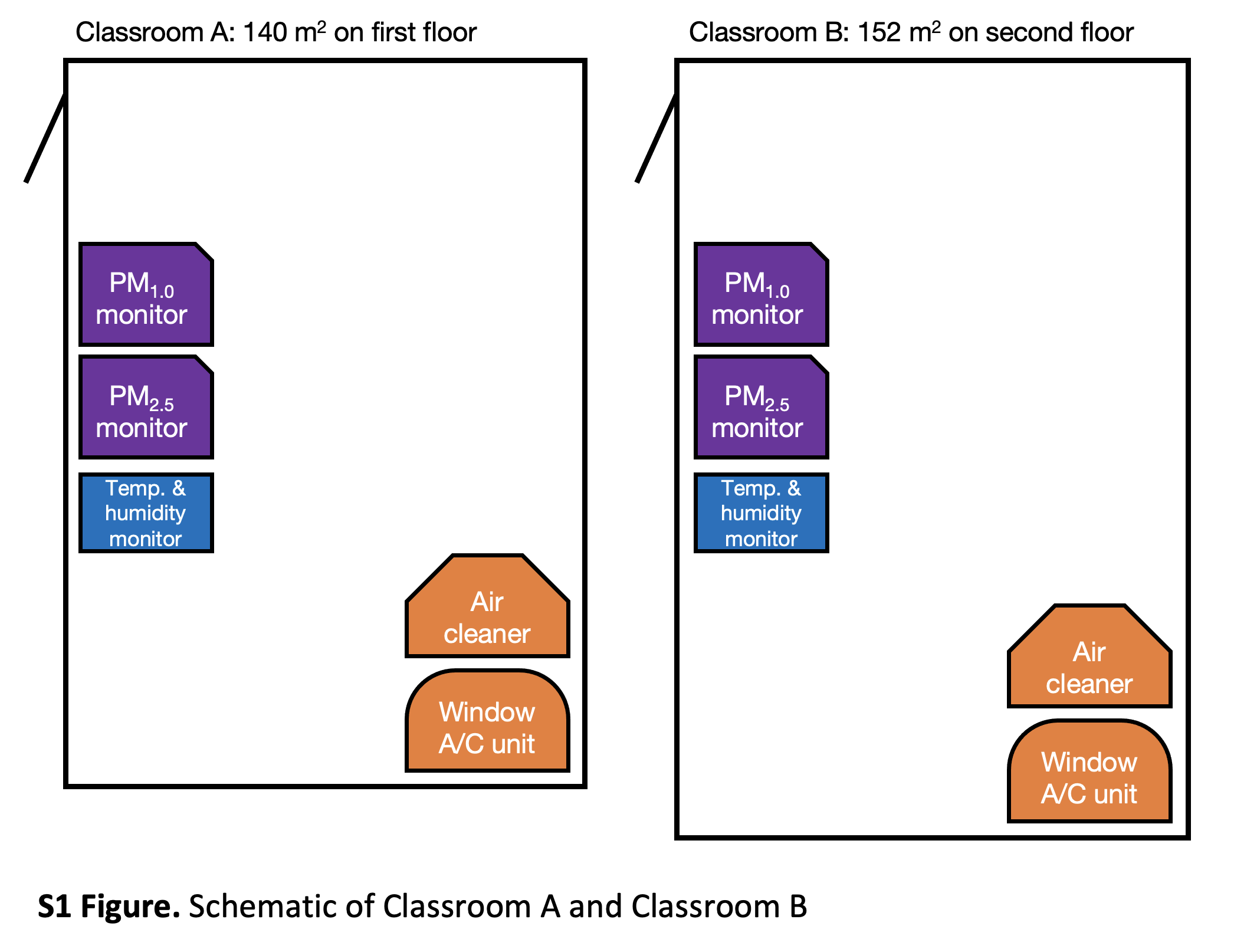

Supplement: S1 Fig — (TIF) [file pone.0278046.s001.tif]
