# Supplementary material for: Effects of portable air cleaners and A/C unit fans on classroom concentrations of particulate matter in a non-urban elementary school
Source: PLoS One. 2022 Dec 1;17(12):e0278046. doi: 10.1371/journal.pone.0278046 (PMC9714748; doi:10.1371/journal.pone.0278046)
Supplement: Attachment — Submitted filename: Revisions Classroom Air Cleaners 8_10_2022.docx [file pone.0278046.s002.docx]

**Response to Editors’ Comments**

**2. You indicated that ethical approval was not necessary for your study. We understand that the framework for ethical oversight requirements for studies of this type may differ depending on the setting and we would appreciate some further clarification regarding your research. Could you please provide further details on why your study is exempt from the need for approval and confirmation from your institutional review board or research ethics committee (e.g., in the form of a letter or email correspondence) that ethics review was not necessary for this study? Please include a copy of the correspondence as an ""Other"" file.**

**Furthermore, we recommend that the consent statement is revised to indicate that the head teachers at the school provided in parentis loco consent to conduct this study.**

***Response:*** Please see the PDF file “IRB Exemption”, categorized as “other”

**3. Thank you for stating the following financial disclosure:**

“Jahred Liddie, a co-author, was supported by a Training Grant in Environmental Epidemiology (T32 E007069) from the National Institute of Environmental Health Sciences. No other specific funding was received for this work by any of the co-authors. Funds from the Department of Environmental Health, Harvard TH Chan School of Public Health and the Harvard Chan NIEHS Center (NIH/NIEHS P30 ES000002) were used to purchase and maintain the equipment used for the study.”

Please state what role the funders took in the study. If the funders had no role, please state: "The funders had no role in study design, data collection and analysis, decision to publish, or preparation of the manuscript."

If this statement is not correct you must amend it as needed.

Please include this amended Role of Funder statement in your cover letter; we will change the online submission form on your behalf.

***Response:*** Thank you for this comment. We have added the provided statement to the funding section of the paper and included a Role of Funder statement in the cover letter.

**4. Thank you for stating the following in the Acknowledgments Section of your manuscript:**

“Jahred Liddie was also supported by a Training Grant in Environmental Epidemiology (T32 E007069) from the National Institute of Environmental Health Sciences. The authors would like to thank Jaime E. Hart and Gary Adamkiewicz for their contributions of expertise and support in this project”

We note that you have provided additional information within the Acknowledgements Section that is not currently declared in your Funding Statement. Please note that funding information should not appear in the Acknowledgments section or other areas of your manuscript. We will only publish funding information present in the Funding Statement section of the online submission form.

Please remove any funding-related text from the manuscript and let us know how you would like to update your Funding Statement. Currently, your Funding Statement reads as follows:

“Jahred Liddie, a co-author, was supported by a Training Grant in Environmental Epidemiology (T32 E007069) from the National Institute of Environmental Health Sciences. No other specific funding was received for this work by any of the co-authors. Funds from the Department of Environmental Health, Harvard TH Chan School of Public Health and the Harvard Chan NIEHS Center (NIH/NIEHS P30 ES000002) were used to purchase and maintain the equipment used for the study.”

Please state what role the funders took in the study. If the funders had no role, please state: "The funders had no role in study design, data collection and analysis, decision to publish, or preparation of the manuscript."

Please include your amended statements within your cover letter; we will change the online submission form on your behalf.

***Response:*** These statements were moved to the funding section.

**5. In your Data Availability statement, you have not specified where the minimal data set underlying the results described in your manuscript can be found. PLOS defines a study's minimal data set as the underlying data used to reach the conclusions drawn in the manuscript and any additional data required to replicate the reported study findings in their entirety. All PLOS journals require that the minimal data set be made fully available. For more information about our data policy, please see http://journals.plos.org/plosone/s/data-availability.**

Upon re-submitting your revised manuscript, please upload your study’s minimal underlying data set as either Supporting Information files or to a stable, public repository and include the relevant URLs, DOIs, or accession numbers within your revised cover letter. For a list of acceptable repositories, please see http://journals.plos.org/plosone/s/data-availability#loc-recommended-repositories. Any potentially identifying patient information must be fully anonymized.

Important: If there are ethical or legal restrictions to sharing your data publicly, please explain these restrictions in detail. Please see our guidelines for more information on what we consider unacceptable restrictions to publicly sharing data: http://journals.plos.org/plosone/s/data-availability#loc-unacceptable-data-access-restrictions. Note that it is not acceptable for the authors to be the sole named individuals responsible for ensuring data access.

We will update your Data Availability statement to reflect the information you provide in your cover letter.

***Response:*** All the data necessary for the analyses have been uploaded. There are three datasets.

**Comments to the Author**

**Reviewer #1: Abstract:**

**The structure of the abstract must be changed. Start with the problem you have found that triggers your research and finalize with the objective, methodology and conclusions.**

**This is the second sentence of the abstract: “Our objective was to determine if use of air cleaners with HEPA filters and air conditioning (A/C) units were associated with changes in particulate matter (PM) air pollution concentrations in a real-world environment.”**

**Could you change the order and include the sentence as the main goal of the article? What is the method to achieve that goal?**

***Response:***

**Abstract (Reorganized)**

Given the increased useing of air cleaners in classrooms during the COVID-19 pandemic as a prevention measure, this study aimed to investigate the the effects of portable air cleaners with HEPA filters and window A/C fans on real-time (1 minute) concentrations of PM less than 2.5 microns (PM_2.5_) or less than 1 microns (PM_1.0_) in two classrooms in a non-urban elementary school in Rhode Island. For half of each school day, settings were randomized to “high” or “low” for the air cleaner and “on” or “off” for the fan. Descriptive statistics and linear mixed models were used to evaluate the impacts of each set of conditions on PM_2.5_ and PM_1.0_ concentrations. The mean half-day concentrations ranged from 3.4 - 4.1 µg/m^3^ for PM_2.5_ and 3.4 - 3.9 µg/m^3^ for PM_1.0_ On average, use of the fan alone decreased PM_2.5_ by 0.53 µg/m^3^ [95% CI: -0.64, -0.42] and use of the filter on high (compared to low) alone decreased PM_2.5_ by 0.10 µg/m^3^ [95% CI: -0.20, 0.005]. For PM_1.0_, use of the fan alone decreased concentrations by 0.18 µg/m^3^ [95% CI: -0.36, -0.01] and use of the filter on high (compared to low) alone decreased concentrations by 0.38 µg/m^3^ [95% CI: -0.55, -0.21]. In general, simultaneous use of the fan and filter on high did not result in additional decreases in PM concentrations compared to the simple addition of each appliance’s individual effect estimates. use of either appliance individually. Our study suggests that concurrent or separate use of an A/C fan and air cleaner in non-urban classrooms with low background PM may reduce classroom PM concentrations.

**Introduction:**

**Lines 101-103: “To the best of our knowledge, this is the first study to provide data on the effectiveness of portable air cleaners and fans operating in tandem to reduce PM2.5 and PM1.0 concentrations in occupied classrooms.” This could be the main objective of the article. I suggest not to mention “To the best of our knowledge, this is the first study…” Just state the objective of the article.**

***Response:***  The objective of this study is to provide data on the effectiveness of portable air cleaners and fans operating in tandem to reduce PM_2.5_ and PM_1.0_ concentrations in occupied classrooms.

**Please, unify the format of the tables.**

***Response:*** We have adjusted the formatting and file types of the tables to be unified.

**Figure 1. Please explain the equation and the parameter “r”.**

***Response:*** We have added a caption within the figure describing both these components.

**Conclusions:**

**Use some of the numerical results to reinforce the statements.**

***Response:*** Please see the tracked changes document that demonstrate the use of numerical results to reinforce the statements.

**Include some limitations you might have found in the methodology and future improvements.**

***Response (bold are the changes):*** This study adds to the limited body of literature surrounding PM_2.5_ and PM_1.0_ concentrations in classroom settings in the United States and provides a foundation for future research in the field. Though we did not consider the effects of seasonality given the small sampling period, seasonality should be considered in future studies. Classroom PM concentrations have been shown to vary by season, with higher concentrations of PM occurring in the winter compared to the summer [32]. Occupant behavior (e.g., window opening) would also vary significantly by season. It would also be worthy to more closely evaluate the impacts of occupancy—the number of teachers and students per classroom—on PM concentrations. Multiple studies have shown that mechanical forces are a primary driver of classroom air quality, therefore it is important to look at how the number of students per classroom influences PM concentrations and the effectiveness of different air quality interventions [32]. **This study only involved two classrooms as a result of availability of equipment and could have been strengthened if more classrooms were evaluated. As a result, the findings from two classrooms in one school limit the generalizability of the study findings even though the study has multiple day measurements.. Furthermore, to better evaluate the effectiveness of air quality intervention, the air quality device should be placed in the same location and at the height in each classroom to ensure consistency in sampling method. Monitoring the CADR levels at different air quality device operating levels would help contextualize the impact of using the air quality devices at different settings. It is also important to note that this study was conducted in a non-urban area with low ambient PM concentrations and is not reflective of how these air quality interventions may work in urban settings or areas with higher ambient PM concentrations.**

**All the data stated in the abstract should have comments in the conclusion section:**

“The mean half-day concentrations ranged from 3.4 - 4.1 µg/m 2 for PM 2.5 and 3.4 - 3.9 µg/m 2 for PM 1.0 On average, use of the fan decreased PM 2.5 by 0.53 µg/m 3 [95% CI: -0.64, -0.42] and use of the filter on high (compared to low) decreased PM 2.5 by 0.10 µg/m 3 [95% CI: -0.20, 0.005]. For PM 1.0 , use of the fan decreased concentrations by 0.18 µg/m 3 [95% CI: -0.36, -0.01] and use of the filter on high (compared to low) decreased concentrations by 0.38 µg/m 3 [95% CI: -0.55, -0.21].”

***Response:*** Thank you - we have added this to the conclusions section.

**Reviewer #2:**

**The manuscript titled ‘Effects of portable air cleaners and A/C unit fans on classroom concentrations of particulate matter in a non-urban elementary school’ documents the effects of air cleaner in the low/high mode and A/C fan in the on/off mode using linear mixed regression models. Interestingly, the manuscript does not discuss potential impacts of students’ activity on classroom PM and on the effects of air cleaner. Because student activity is an important factor affecting classroom PM concentrations and effect of air cleaners on classroom PM, this needs to be discussed in Discussion. The manuscript may also consider the following comments to improve clarity and flow of the manuscript.**

***Response:*** Thank you – we have added not being able to record student activity (due to COVID-19 limitations at the time) as a limitation to the study, which can be found in the Discussion. It should be noted that we asked the teachers to track times of events that may impact PM concentrations (e.g. bus arrivals, times during which all students typically leave the classroom), but the data was not sufficient to make any conclusions.

**Major comments:**

**1. Lines 44-47: It should be mentioned that the effect of fan was adjusted for air cleaner; likewise, the effect of air cleaner was adjusted for A/C fan.**

***Response:*** Thank you – please see lines 40-46 in the tracked changes document to see the clarification on the adjustments for air clear and the A/C fan.

**2. Lines 48-51: General statement (lines 48-49) of the study findings and the conclusion (lines 50-51) don’t seem to agree in the current writing. It looks like that the conclusion should be modified to more specific one because the effects of concurrent use of an A/C fan and air cleaner on reducing the PM were dependent of the mode of the air cleaner and PM size. The proper conclusion would have implication in saving energy.**

***Response:*** Simultaneous use of the fan and air cleaner did not result in significant, additional decreases in PM2.5 - as stated in the results section, the interaction term was actually significantly positive. Regarding PM1.0, no significant differences associated with simultaneous use of the air cleaner and fan were found. We have updated the phrasing of the results in the Abstract as well as in the Results section to better reflect this finding.

**3. Lines 48-49: Does the ‘additional decreases in PM concentrations’ mean no interaction effect between fan on and air cleaner on high? But the interaction was only significant for PM2.5 but not for PM1.0. If this statement was from the interaction model outputs, it should be also specific to PM size.**

***Response****:* Thank you for this comment. As clarification and as described in the Result section, the interaction term was significantly for PM2.5 and null for PM1.0. This indicated that concurrent use of the appliances, in our study setting, did not result in significantly lower PM concentrations for either size fraction below the simple addition of each separate effect estimates. In line with previous comment, we have rephrased the results description in the Abstract and Result sections to reflect this more clearly.

**4. Lines 79-84: The sentences are about health effects of PM, which has nothing to do with use of air cleaner or improved ventilation that seemed to be the theme for the previous sentences within the paragraph. Thus, it would flow better if these were moved to the next paragraph (after the last sentence of the next paragraph that is describing PM health effect).**

***Response:*** Given the challenges of installing new HVAC systems or filters in school buildings, portable air cleaners and fans have become popular, less expensive alternatives to reduce PM concentrations. Air cleaners with high-efficiency particulate air (HEPA) filters, which are at least 99.97% efficient at capturing particles 0.3 µm and larger in size, offer the possibility of substantially reducing SARS-CoV-2 viral particles as well as improving overall IAQ [7,5]. Beyond the current context of the COVID-19 pandemic, improved air ventilation is associated with lower school absenteeism, better performance on cognitive function tests, and fewer respiratory symptoms, such as those related to asthma, lung inflammation, and allergies [9, 10, 11]. PM_2.5_ exposure in children has been associated with asthma incidence (OR=1.10, 95% CI:1.01, 1.20), prevalence of asthma symptoms (OR=1.08, 95% CI:1.02, 1.16), and rhinitis (OR=1.15, 95% CI: 1.05,1.26) [12]. A review study of 33 articles further provided evidence that exposure to particulate air pollution has adverse impacts on children’s respiratory health, with stronger negative effects seen among children with asthma [13].

PM is one of the most common pollutants that could potentially degrade air quality in classrooms [14]. Indoor PM levels are influenced by several factors, such as ambient air pollution levels, air exchange rates, occupancy, type and intensity of indoor activities, and particle sizes [14, 15, 16]. One study in Munich, Germany found that PM concentrations in classrooms are about six times higher than outdoor concentrations [17]. Children are particularly vulnerable to potential health consequences related to PM exposure due to their immature respiratory and immune systems and greater breathing rates per body weight [11]. **Beyond the current context of the COVID-19 pandemic, improved air ventilation is associated with lower school absenteeism, better performance on cognitive function tests, and fewer respiratory symptoms, such as those related to asthma, lung inflammation, and allergies [9, 10, 11]. PM_2.5_ exposure in children has been associated with asthma incidence (OR=1.10, 95% CI:1.01, 1.20), prevalence of asthma symptoms (OR=1.08, 95% CI:1.02, 1.16), and rhinitis (OR=1.15, 95% CI: 1.05,1.26) [12]. A review study of 33 articles further provided evidence that exposure to particulate air pollution has adverse impacts on children’s respiratory health, with stronger negative effects seen among children with asthma [13].**

**5. Wondering if the authors examined interaction between classroom humidity and fan or between humidity and air cleaner in the regression models?**

***Response:*** We tried this and a model with a three-way interaction - generally, the main effects are almost identical, although there are some significant but small interactions with humidity that vary for the size fractions. However, we decided not to include the result due the difficulty to interpret the impact and the concern that if may distract from results.

**6. Lines 198-205: It seemed that fan effect was bigger for PM2.5 then PM1.0, but air cleaner effect was bigger for PM1.0 than PM2.5. Isn’t this worth to discuss further in Discussion?**

***Response:*** Thank you - this point has been addressed in the Discussion.

**7. The title of Table 2 should state ‘Unadjusted ANOVA’ if the ANOVA models were not adjusted for anything.**

***Response:*** Thank you - we have added this distinction.

**8. Lines 235-243: The studies discussed are all home studies. Are there any studies of evaluating the effect of air cleaners in classrooms? Unless discussion is strictly limited to HEPA air cleaners, there are some studies examining effects of air cleaners in classrooms (e.g., Wargocki et al., HVAC R Res. 14 (2008): 327-344; Mattsson and Hygge, 2005 Indoor Air Conference Proceedings, pp 1111-1115; Park et al., Building and Environment 167, 2020: 106437), which should be discussed.**

***Response:*** The studies discussed were limited to HEPA air cleaners. We discussed one classroom study that also used HEPA air cleaners, but acknowledged a general lack of existing literature. However, we have cited more school studies elsewhere in the Discussion, including a few mentioned by the reviewer.

**9. Lines 256-257: The discussion doesn’t agree with the current study finding. It was reported in Results that the air cleaner on high mode reduced PM1.0 concentrations more than PM2.5. Thus, your argument in this sentence about ‘less effective at removing particles for smaller size PM by air cleaner on high mode’ is not supported by your own finding.**

***Response:*** Thank you for this comment. We went to this paragraph and revised our language. We had incorrectly discussed particle size instead of particle concentration since our study was in a classroom that had very low PM concentrations. We also added additional context. Please see lines 365- 398 (in track changes document).

**10. Line 268: ‘….some average daily ambient …’. Specify ‘some’ in this sentence because there are only two days when ambient PM2.5 concentrations were lower than classroom PM2.5.**

***Response:*** Thank you - we have added this clarification.

**11. Lines 261-273: This paragraph was not clear. What is the main discussion point of the paragraph? The paragraph may need to be modified for clarification.**

***Response:*** Thank you – The main discussion of the paragraph was to highlight the fact that the study was conducted in a location that has very low ambient PM concentrations and that those low ambient concentrations may have impacted classroom PM concentrations. We edited the paragraph starting at line 404-422 (in the track changes document) to emphasize the low ambient PM concentrations.

**12. In the limitation section, the small number of classrooms in the study also needs to be mentioned as a limitation. Findings from only two classrooms in one school may not be generalizable although the study has multiple day measurements.**

***Response (bold are the changes):*** This study adds to the limited body of literature surrounding PM_2.5_ and PM_1.0_ concentrations in classroom settings in the United States and provides a foundation for future research in the field. Though we did not consider the effects of seasonality given the small sampling period, seasonality should be considered in future studies. Classroom PM concentrations have been shown to vary by season, with higher concentrations of PM occurring in the winter compared to the summer [32]. Occupant behavior (e.g., window opening) would also vary significantly by season. It would also be worthy to more closely evaluate the impacts of occupancy—the number of teachers and students per classroom—on PM concentrations. Multiple studies have shown that mechanical forces are a primary driver of classroom air quality, therefore it is important to look at how the number of students per classroom influences PM concentrations and the effectiveness of different air quality interventions [32]. **This study only involved two classrooms as a result of availability of equipment and could have been strengthened if more classrooms were evaluated. As a result, the findings from two classrooms in one school limit the generalizability of the study findings even though the study has multiple day measurements.. Furthermore, to better evaluate the effectiveness of air quality intervention, the air quality device should be placed in the same location and at the height in each classroom to ensure consistency in sampling method. Monitoring the CADR levels at different air quality device operating levels would help contextualize the impact of using the air quality devices at different settings. It is also important to note that this study was conducted in a non-urban area with low ambient PM concentrations and is not reflective of how these air quality interventions may work in urban settings or areas with higher ambient PM concentrations.**

**Reviewer #3:** Dear Author,

**Your manuscript follows a very interesting approach. However, important information is missing to be able to assess the results and evaluate them for a school. For example, a room sketch is missing, as well as information about the ceiling height, with the coordinates for the air purifier, A/C unit and also the measuring points. Furthermore, you do not address the CADR values that are realized at different levels of the air cleaner. The sound level at the different levels is also not mentioned, although this is a critical factor for use in classrooms. The same applies to the A/C unit. I read online that a sound pressure level of 57 dB(A) is generated at the "Low" level, which is clearly too high and would in turn have an impact on the students' ability to concentrate. In my view, when you address the specific case in schools, you have to take these points into account.**

***Response:*** We added sound level as a limitation in our discussion. We were concerned about the impact of the sound in the classroom; however, the air purifiers and A/C fans were not so loud and did not interfere with the ability to teach in the classroom. Furthermore, internet research indicates that 60 dB is conversation volume. We currently do not have a sketch of the classrooms but we included dimensions of the classrooms and location of the air purifiers in the classroom in the methods section.
